# Face in collision: emotional looming stimuli modulate interpersonal space across development and gender

**DOI:** 10.1007/s00426-021-01590-7

**Published:** 2021-09-29

**Authors:** Valentina Silvestri, Massimo Grassi, Elena Nava

**Affiliations:** 1 Department of Psychology, University of Milan-Bicocca, Piazza dell’Ateneo Nuovo 1, 20126 Milan, Italy; 2 Department of Psychology, University of Padua, Via Venezia 8, 35129 Padua, Italy

## Abstract

Basic visual functions have evolved to allow for rapid detection of dynamic stimuli in our surrounding environment. In particular, looming stimuli are of relevance because they are expected to enter the individual’s interpersonal space representing a potential threat. Different studies showed that emotions can modulate the perception of visual looming stimuli and the borders of interpersonal space, defined as the area around the body that individuals maintain between themselves and others during social interactions. Here, we investigated how emotions modulate the perception and the physiological correlates of interpersonal space and whether such indexes change across age and gender. Children and adults were asked to quickly react to emotional looming stimuli while measuring their skin conductance response (SCR). We found that emotional looming stimuli shrink the borders of interpersonal space of males more than females, and that this pattern does not change with age. In addition, adults reacted faster to angry than happy and neutral faces, which is in line with the notion that threatening stimuli capture attention more quickly than other types of emotional stimuli. However, this was not observed in children, suggesting that experience with negative stimuli, rather than the evolutionary meaning they possess, may influence the boundaries of interpersonal space. Overall, our study suggests that interpersonal space is modulated by emotions, but this appears to be modulated by gender and age: while behavioural responses to emotional looming stimuli refine with age, physiological responses are adult-like as early as 5 years of age.

## Introduction

The space around our bodies represents an important and vital area. Indeed, we closely monitor the objects that enter this area, as well as monitor the actions performed by our limbs that interact with objects in the outer world. Importantly, one of the most important and meaningful interactions we have both in close and far proximity of our bodily space is with other human beings. However, social encounters are not always welcome, and the space around the body has been therefore defined as an “area individuals maintain around themselves into which others cannot intrude without arousing discomfort or even withdrawal” (Hayduk, 1983). The concept of “interpersonal space” (Candini et al., 2019; D'Angelo et al., 2019; Iachini et al., 2014; Patané et al., 2017) emphasises the importance of emotions in the construction of this space, and should not confuse with the concept of “peripersonal space”, which is an “action space”, conceptualised as a multisensory interface, which detects and predicts potential interactions between the body and the environment in order to generate suitable motor outcomes (Brozzoli et al., 2013; Iachini et al., 2014; Rizzolatti et al., 1981).

Nevertheless, both peripersonal and interpersonal spaces appear to share common mechanisms and features, and to be influenced by different contextual factors, the most important of which appears to be the emotional valence of the stimuli presented (Ferri et al., 2015; Vagnoni et al., 2012, 2015). A typical way to assess the role of emotions in modulating the space around our bodies is to use looming stimuli. These stimuli are known to be perceptually salient stimuli per se, as they are moving stimuli and strongly capture attention (Neuhoff, 1998; Grassi, 2010; Grassi & Pavan, 2012; Grassi & Mioni, 2020). For example, Ferri and colleagues (2015) showed that emotion-inducing looming sounds (e.g. sounds inducing negative feelings) modulated the boundaries of participants’ peripersonal space, in that negative sounds triggered faster responses to short tactile stimuli applied to the right hand of the participants (Ferri et al., 2015). In a similar vein, Vagnoni and colleagues (2012) found that threatening visual stimuli, such as snakes or spiders, induced participants to misperceive the time-to-collision to their body with respect to non-threatening stimuli, such as butterflies and rabbits. In a further study, the same authors (2015) investigated the visual-evoked potentials (VEPs) and the oscillatory neural responses measured with electroencephalography to threatening stimuli approaching and found a neural modulation by the emotion and the speed of the stimulus, particularly revealing a disruption of synchronisation in sensorimotor areas, known to be activated by looming stimuli.

As shown by Ellena and colleagues (2020), fearful (looming) stimuli also modulate the distribution of spatial attention: participants had to respond to tactile stimuli at their cheeks while watching a virtual reality looming avatar face that displayed a joyful or fearful face. To assess the role of spatial attention, a ball was presented at trial onset either close or far from the avatar’s face.

Responses to tactile stimuli were facilitated by both facial expressions, but while joyful faces facilitated tactile stimulus detection without redistributing spatial attention, fearful faces redirected attention towards the periphery, likely promoting the protective function of the space around the body.

For example, in a study using immersive virtual reality, Ruggiero and colleagues (2017) asked participants to establish a comfort distance (‘interpersonal space’) and a reachability distance (‘peripersonal space’) from avatars expressing either happy, angry or neutral emotions. Both peripersonal and interpersonal spaces were influenced by emotions, with negative emotions ‘expanding’ the space more than the neutral faces, suggesting that space, emotional valence and action, all interact in order to prepare an adequate motor output. These results suggest that the social domain of interpersonal space shares common mechanisms to the sensorimotor domain of peripersonal space.

Emotions, as conveyed through facial expressions, can also alter the social comfort space at a physiological level: Cartaud and colleagues (2018) exposed participants to neutral, angry or happy facial expressions presented at the perceptual threshold asking subsequent judgment of interpersonal comfort distance while electrodermal activity (EDA) was registered. The authors showed that the electrodermal activity increased when participants were previously exposed to barely visible angry faces, but only when the stimulus violated the participant’s interpersonal comfort space. That is, negative/threatening emotions were perceived as such, only if they ‘passed’ the border of interpersonal/comfort space.

Other factors appear to influence the perception of interpersonal space, these being gender and age (Cléry et al., 2015; Iachini et al., 2016; Lloyd, 2009). Iachini and colleagues (2016) asked participants to judge the distance between themselves and male or female virtual humans of different ages (i.e. children, young adults and older adults). Participants maintained a greater distance to males compared to females, and, in particular, male participants maintained a shorter distance from the female avatar. Furthermore, women, but not men, maintained a shorter distance from children than adults and older avatars, suggesting that children represent—at least for women – an important cue associated with specific responses and positive approach reactions (Senese et al., 2013; Iachini et al., 2016).

Gender differences have also been found in neuroimaging studies. Females perceive approaching stimuli, specifically if male faces, as more intruding and potentially threatening, as evidenced by higher amygdala activity (Wabnegger et al., 2016). In particular, the amygdala appears to regulate interpersonal distance in humans (Kennedy et al., 2009), and its activity correlates with greater preferred distances to different emotions, e.g. preferred distance from angry faces is associated with higher activity in the amygdala (Vieira et al., 2017).

Finally, interpersonal distance appears to increase throughout development (Aiello & Aiello, 1974). However, to date only a few studies have addressed this issue. In particular, studies using looming stimuli to investigate sensitivity to approaching stimuli in development have shown that newborns can discriminate between the trajectories of moving stimuli, displaying a preference for stimuli directed towards their bodies than stimuli moving away from their bodies (Orioli et al., 2018). Infants between 2 and 11 weeks of age also possess perceptual capacities to quickly detect some qualities of the approaching object, such as distance and direction of impact (Ball & Tronick, 1971). Thus, these studies suggest that sensitivity to looming approaching stimuli may possess an evolutionary meaning, and thus be in place long before an individual can decide which action to take (see Neuhoff, 1998).

Indeed, earlier studies investigating interpersonal distance in children have shown that in ecological settings, older children tend to keep more distance from their peers and acquire adult’s proxemic behaviour by age 12 (Aiello & Aiello, 1974). In particular, while children between 6 and 12 tend to keep less distance from their peers, irrespective of gender, between 12 and 16 males tend to keep more distance from their peers in comparison to females. The fact that gender differences start influencing proxemic behaviour only in early adolescence suggests social learning (i.e. learning of social norms, by which males maintain more distance from other people).

This pattern was corroborated by Wesley and McMurphy (1982), who measured the patterns of distances maintained between children and their caregivers, and children and their peers during freeplay. The authors found that children’s distances towards the adults increased with age, while the proximity towards peers increased with age, suggesting that the type of social relationship modulates interpersonal distance.

To date, the only study conducted with older children using looming stimuli has shown that until age 15, children underestimate the velocity of approaching vehicles (Wann et al., 2011; Purcell et al., 2012), thus placing them at risk of being hit when crossing a street. Although this study does not suggest that sensitivity to looming decreases across early and late development, it certainly reveals that older children take in consideration several cues in order to evaluate the looming stimuli (i.e. rate of expansion of the stimuli, time needed to cross the street). This, in turn, may slow down their evaluation of the approaching stimuli.

Given the lack of studies in the developmental population, in the current study, we aimed to investigate how emotions modulate the perception and the physiological correlates of interpersonal space across age and gender, by presenting children and adults with emotional looming stimuli and by assessing their skin conductance responses. We predicted that if detecting early negative emotions is generally advantageous, the autonomous nervous system should detect early this type of stimulus independently of age. Furthermore, if skin conductance anticipates and influences behaviour, we should observe an association between physiological response and behaviour as in Cartaud et al., (2018, 2020).

The use of skin conductance was motivated by several reasons: first, this method is particularly suited to assess the unconscious activity related to emotional processing (Khalfa et al., 2002; Nakasone et al., 2005); second, skin conductance responses commonly correlate with activity in the amygdala (Laine et al., 2009; Wood et al., 2014), which has been found to regulate interpersonal space perception (Kennedy et al., 2009). Finally, skin conductance represents a non-invasive, easy and reliable method to assess autonomic responses in children of all ages (Crone & Van der Molen, 2007; Nava et al., 2016).

## Methods

Participants were thirty right-handed students (fifteen females) aged 19–40 years (*M* = 24; SD = 3.64), recruited at the University of Milan-Bicocca in exchange for course credit, and thirty children (fifteen females) aged four to six years (*M* = 5.13; SD = 0.43). Five additional children were tested but were excluded because they did not complete the experiment (*n* = 3) or did not understand the task (*n* = 2).

Children were recruited through a database at the Department of Psychology (University of Milan-Bicocca), in which the contacts of the parents of children are stored (i.e. parents whose children previously participated in other experiments and expressed the consent to participate in other experiments). The sample was determined through a priori power analysis and data were not analysed until data collection was completed. A power analysis for a repeated measure ANOVA with within–between interaction with two factors (age of the stimuli and emotions) revealed that 60 participants would be needed to have a 90% of chance to observe a significant effect with an alpha level of 0.05 and a medium effect size.

All participants had normal or corrected-to-normal visual acuity and were all right handed. The parents of the children signed a written informed consent before the children were tested. The adult participants also gave their written informed consent prior to take part in this study. This study was approved by the local Ethics Committee (Prot. No. 391, 23/07/2018), in line with the WMA Declaration of Helsinki for research involving human subjects.

### Apparatus, materials and stimuli

The stimuli were presented on a 24” Lenovo monitor and the participants sat at ~ 45 cm from the screen.

**Fig. 1:**
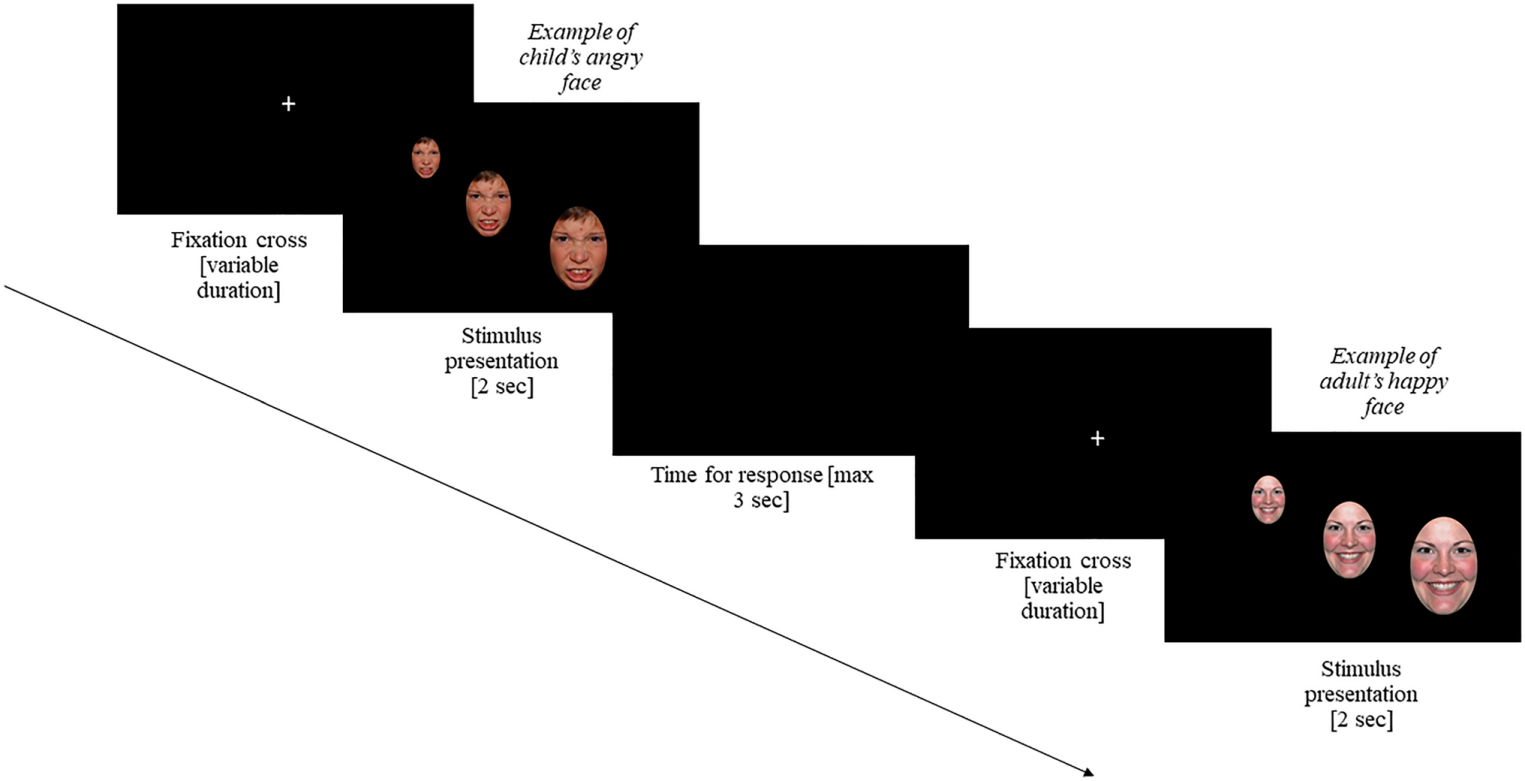
Example of stimuli and procedure

A schematic representation of the stimuli is presented in Fig. 1. The stimuli consisted of adult and children faces of both sexes, selected from the "Nim Stim Face Stimulus Set" (Tottenham et al., 2009) and the "NIMH Child Emotional Faces Picture Set" (Egger et al., 2011), respectively. From each dataset, we selected four male and four female identities, each presenting an angry, neutral or happy facial expression. An oval mask was superimposed on each image, so that all stimuli were equal in size. There were a total of 48 faces (2 × gender, 2 × ages, 3 × facial expressions), presented in two blocks, for a total of 96 trials.

In order to simulate the motion of a looming object, the stimulus increased exponentially in size over 2 s. This simulates the proximal stimulation pattern produced by the motion of an object moving at a constant speed towards a perceiver. Such an object generates a change in retinal size that is exponential (i.e. linear on a logarithmic scale). In the current experiment, the minimum stimulus size was 3° (visual angle) and the maximum stimulus size was 36°.

#### Skin conductance

The electrodermal activity was measured using a biological signal amplifier (MP150, Biopac Systems, Inc). The amplifier was connected to the computer via optical connection. The signal acquisition parameter was set at 5 μmho/V and the signal was sampled at 100 Hz. The recording and analysis of the skin conductance response (SCR) was carried out using the AcqKnowledge Software provided by Biopac System, using the peak-to-peak index to measure the variations in the phasic phase of the skin conductance.

Specifically, the peak-to-peak refers to the amplitude of the signal recorded within each single trial, calculated as the difference between the highest and the lowest peak of each single trial. The skin conductance signal was acquired by applying two electrodes to the index and ring finger of the participant's left hand, while the reference electrode was applied to the forearm.

### Procedure

Participants were tested individually in a quiet room of the University of Milano-Bicocca.

Skin conductance electrodes were attached to the participants’ fingers first, then participants were sat in front of a computer screen displaying a fixation point in the centre. Each trial was manually started by the experimenter, who pressed on the spacebar of the computer and concurrently placed a timestamp on the EDA signal. From this timestamp, we then calculated a 6-s epoch from which we calculated the peak-to-peak of each participant. The intertrial interval was approximately 8 s but could vary depending on the participant’s attention. This is particularly useful with children, to ensure that they are attending the stimulus once it is launched. The task was similar to the comfort-distance judgments task of Iachini et al. (2014), and participants were instructed to respond as fast as possible to the approaching faces (by pressing the left mouse button) when they felt that the stimuli was entering their comfort zone. In particular, all participants were instructed to press the button as soon as they felt that the distance between them and the face approaching was making them uncomfortable.

The stimulus disappeared following mouse button press or after a maximum of 3 s following stimulus onset. All participants were allowed to take a break between one block and the other.

At the end of the experiment, participants were given a questionnaire in which they had to evaluate how angry the face looked on a Likert scale ranging from 0 to 4, in which 0 indicated "Not at all angry", while 4 indicated "Very angry". This means that if participants were able to discriminate the facial expressions correctly, they would report 0 when presented with happy faces, and higher ratings when presented with angry faces. The questionnaire was administered to exclude that any potential difference between children and adults could be the result of a different ability in emotion discrimination.

## Results

### Skin conductance

For each participant, mean peak-to-peak responses within a time window of 6 s were extracted from each trial. Trials containing movement artefacts, as observed from the raw data, were manually discarded. Because the data violated the test for homogeneity of variance (Levene’s test), data were log-transformed using the formula log(*x* + 1), fulfilling the assumption of homogeneity (*p* > 0.2).

**Fig. 2:**
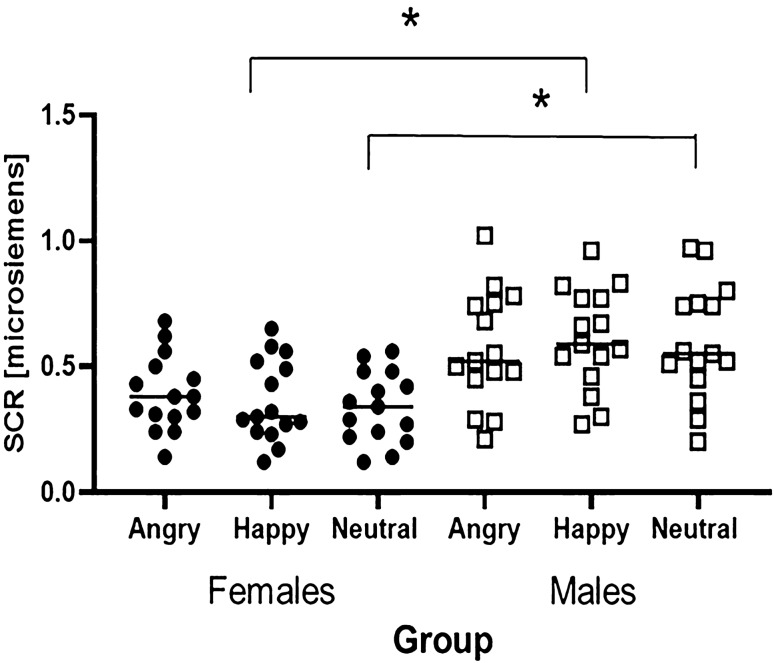
Distribution of SC responses for females and males, irrespective of age. Error bars represent 95% confidence intervals of the mean

The transformed data were entered in a repeated measures ANOVA, with Age of Stimuli (children and adult images), Gender of Stimuli (female and male images) and Emotion (happy, angry, fearful) as within-subjects factor, and Group (children and adult participants) and Gender (male and female participants) as between-subjects factor. The analysis revealed a significant main effect of Group, *F*(1, 56) = 26.53, *p* < 0.001, partial *η*^2^ = 0.32, due to children’s (*M* = 0.21, SD = 0.10) overall higher arousal compared to adults (*M* = 0.11, SD = 0.08). There was also a main effect of Gender, *F*(1, 56) = 11.52, *p* = 0.001, partial *η*^2^ = 0.17, due to females (*M* = 0.13, SD = 0.08) showing an overall lower arousal than males (*M* = 0.19, SD = 0.11). This main effect was qualified by a significant Emotion × Gender interaction, *F*(2, 112) = 5.57, *p* = 0.005, partial *η*^2^ = 0.09. Bonferroni corrected post hoc tests revealed that males displayed higher arousal than females for happy (males: *M* = 0.19, SD = 0.09; females: *M* = 0.13, SD = 0.08, *p* = 0.008) and neutral faces (males: *M* = 0.19, SD = 0.10; females: *M* = 0.12, SD = 0.07, *p* = 0.006); however, females and males did not differ for angry faces (*p* = 0.22, see Fig. 2). No other significant interaction, referring to developmental changes, occurred (Emotion x Group: *p* = 0.73; Age of Stimuli × Group: *p* = 0.75; Group × Gender of Stimuli:  *p* =  0.76).

### Reaction times

To assess whether different emotions modulate the interpersonal space on a more explicit level, we conducted the same analyses as for the SC responses, by entering the mean reaction times in a repeated measures ANOVA, with Age of Stimuli, Gender of Stimuli and Emotion, as within-subjects factor, and Group and Gender as between-subjects factor.

There was a main effect of age of stimuli, *F*(1,56) = 8.38, *p* = 0.005, partial *η*^2^ = 0.13, due to faster reaction times of all participants when the presented face was an adult (*M* = 1602 ms, SD = 407) than a child (*M* = 1646 ms, SD = 396). There was also a main effect of Emotion, *F*(2,112) = 11.25, *p* < 0.001, partial *η*^2^ = 0.17, due to participants having faster reaction times for angry (*M* = 1581 ms, SD = 413) rather than happy (*M* = 1660 ms, SD = 386) or neutral faces (*M* = 1629 ms, SD = 405). We found a main effect of Gender, *F*(1,56) = 4.11, *p* = 0.047, partial *η*^2^ = 0.07, due to males (*M* = 1531, SD = 409) having faster reaction times than females (*M* = 1715, SD = 374).

**Fig. 3:**
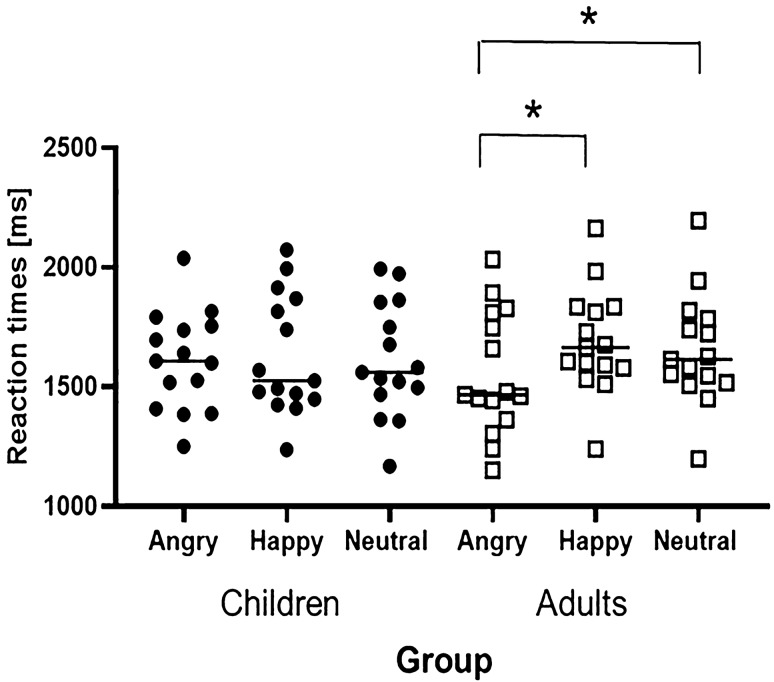
Distribution of reaction times on each emotion for children and adults, irrespective of Gender. Error bars represent 95% confidence intervals of the mean

We observed a significant Emotion × Group interaction, *F*(2,112) = 6.11, *p* = 0.003, partial *η*^2^ = 0.09, due to adults responding faster to angry (*M* = 1556, SD = 450) than happy (*M* = 1691, SD = 373, *p* < 0.001) and neutral faces (*M* = 1654, SD = 399, *p* = 0.001). It is noted that happy and neutral did not differ (*p* = 0.99, see Fig. 3).

Two further triple interactions emerged: Group × Gender × Gender of Stimuli, *F*(1,56) = 4.80, *p* = 0.03, partial *η*^2^ = 0.08 and a marginally significant Emotion × Gender × Gender of Stimuli, *F*(2,112) = 3.18, *p* = 0.05, partial *η*^2^ = 0.05. In particular, the first triple interaction revealed that seeing stimuli of same or different gender with respect to own gender did not change across age when males saw either a same-sex (adults: *M* = 1605, SD = 435; children: *M* = 1430, SD = 318, *p* = 0.87) or a different-sex face (adults: *M* = 1619, SD = 416; children: *M* = 1470, SD = 305, *p* = 0.94), and when females saw either a same-sex (adults: *M* = 1669, SD = 371; children: *M* = 1755, SD = 307, *p* = 0.99) or a different-sex face (adults: *M* = 1640, SD = 379; children: *M* = 1797, SD = 273, *p* = 0.93). It is noted that no other relevant comparison proved significant from this interaction (all *p* > 0.10).

The second triple interaction revealed that seeing stimuli of same or different gender with respect to own gender did not modulate the response across age, irrespective of whether the emotion was angry in same-sex (females: *M* = 1636, SD = 382; males: *M* = 1502, SD = 409, *p* = 0.96) or different-sex (females: *M* = 1663, SD = 408; males: *M* = 1519, SD = 374, *p* = 0.93), happy in same-sex (females: *M* = 1758, SD = 330; males: *M* = 1513, SD = 378, *p* = 0.31) or different-sex (females: *M* = 1777, SD = 311; males: *M* = 1594, SD = 387, *p* = 0.73), or neutral in same-sex (females: *M* = 1741, SD = 342; males: *M* = 1538, SD = 397, *p* = 0.47) or different-sex (females: *M* = 1717, SD = 343; males: *M* = 1520, SD = 381, *p* = 0.64).

Finally, to observe whether autonomic responses can predict behaviour, we entered skin conductance responses for angry, happy and neutral faces as predictor in a regression analysis, with reaction times as dependent variable, separately for age group and gender. However, none of the regressions proved significant (all *p* > 0.5). We also checked for any possible correlations between behaviour and implicit response, and even though all the relations had a positive trend, we did not find any significant correlations (all *p* > 0.12).

Lastly, the self-report questionnaire that concluded the experiment revealed that children did not differ from adults in the way they judged the angriness of the faces (*Z* = − 0.17, *p* = 0.86, Mann–Whitney test). All data are available at the following link: https://osf.io/nur5q/.

## Discussion

The present study investigated whether the emotional value of looming faces modulates the perception and arousal of adults and children, and whether this is influenced by the gender of the participant and the age of the looming face. We found a series of findings, the most important of which regards the modulating role of age and gender, depending on type of measure assessed (i.e. physiological vs behavioural response).

The physiological results showed that males, irrespective of age of the participant, displayed higher arousal when the approaching emotion was happy and neutral with respect to females, but not when it was angry. This pattern appears partially at odds with previous studies. Indeed, some studies have shown that women are more susceptible to emotional context, as revealed by skin conductance and neuroimaging findings. For example, Bianchin and Angrilli (2012) found a greater startle reflex as well as a larger slow evoked potential in women than men when presented with unpleasant and stressful stimuli. It is noted that Wrase and colleagues (2003) found no difference in skin conductance response across gender when participants were presented with emotional pictures. However, at a more central level, using functional magnetic resonance (fMRI), they found that men recruited the amygdala more than women when the content of the images was pleasant, while women recruited the inferior and medial frontal gyrus when presented with unpleasant images.

Importantly, our skin conductance data suggest that, even though children displayed overall higher SCR, emotional stimuli did not influence the responses across age. Thus, our physiological data suggest that by the preschool years, children’s interpersonal space is similarly influenced by emotions as in adults, and that gender already plays a modulating role by age 5.

Interestingly, gender differences influenced arousal, but not behaviour.

On the contrary, analyses conducted on reaction times revealed a developmental change, by which adults were faster when presented with angry with respect to both happy and neutral faces, but children’s reactivity was not influenced by type of emotion, that is, they responded equally fast to angry, happy and neutral faces. The fact that only adults reacted faster to angry faces suggest that the advantage for negative stimuli may be the result of experience. That is, at least on a behavioural level, seeing an angry face approaching shrinks the interpersonal space of adults, but not children.

The absence of difference in reaction times for emotions in children could depend upon a methodological limitation. Indeed, different studies have shown that there are great individual differences in reaction times in young children, with standard deviations that may prevent any significant difference to emerge (Bucsuházy & Semela, 2017; Lange-Küttner, 2012). Moreover, children show overall longer reaction times than adults, likely because of less motor control and attention. Thus, any comparison between children and adults should be taken with caution (Bucsuházy & Semela, 2017; Lange-Küttner, 2012), and this may well apply to our study too.

Finally, it should be discussed why we found a dissociation between physiological and behavioural data, which is at odds with other studies (Cartaud et al., 2018). In particular, we found a dissociation between behaviour and physiology for the age of the face presented: all participants responded faster to adult than child faces, but this was not evident at a physiological level. The fact that adult faces influenced more the interpersonal space of participants is in line with previous studies that have observed an expansion of own comfort space when the approaching face was an adult over a child face (Iachini et al., 2014), likely because children are perceived as less threatening than adults. The fact that also children responded faster to adult than children faces could be explained by the unfamiliarity of the stimuli. Indeed, children not only prefer familiar over unfamiliar faces (Bushnell, 2001; Richter et al., 2016) but also tend to trust and approach familiar over unfamiliar faces (Rotenberg, 2010; Richter et al., 2016).

In conclusion, our study has shown that the boundaries of interpersonal space are modulated by emotions and that this modulation appears to be constrained by gender and age. In particular, our results have shown that physiological responses are modulated by gender, whereas behavioural responses are modulated by age.
